# Collection calendar: the diversity and local knowledge of wild edible plants used by Chenthang Sherpa people to treat seasonal food shortages in Tibet, China

**DOI:** 10.1186/s13002-021-00464-x

**Published:** 2021-06-10

**Authors:** Xiao-Yong Ding, Yu Zhang, Lu Wang, Hui-Fu Zhuang, Wen-Yun Chen, Yu-Hua Wang

**Affiliations:** 1 Yunnan Key Laboratory for Wild Plant Resources, Kunming Institute of Botany, Chinese Academy of Sciences, Kunming, China; 2 University of Chinese Academy of Sciences, Beijing, China

**Keywords:** Wild edible plants, Local knowledge, Seasonal food shortage, Sherpa, Chenthang Town, Everest region

## Abstract

**Background:**

Wild edible plants (WEPs) are non-cultivated and non-domesticated plants used for food. WEPs provided food, nutrition, herbs and other plant products for people in underdeveloped areas, such as the Everest region, to maintain their daily lives. Chenthang Town is the only Sherpa ethnic township in Tibet, China. The core purpose of this research is to investigate, collect and record the WEPs and related local knowledge and functions within the Sherpa community. The ultimate goal is to answer the question of why Sherpa people choose these particular plants.

**Materials and methods:**

The field study was carried out in the six Sherpa communities of Chenthang Township from September 2019 to August 2020. The WEPs and related local knowledge were collected through semistructured interviews and direct observations. The field work was performed with the assistance of local guides. During the field survey, we collected plant specimens based on the principle of one plant with one vernacular name. In this study, we utilised a use report (UR) and cultural importance index (CI) to evaluate the comprehensive utilization value of WEPs in the daily diet of Sherpa people.

**Results:**

We interviewed 78 people individually who provided us with 1199 use reports. In total, we collected 84 WEPs belonging to 65 genera in 41 families. These species were identified as 78 distinct ethno-species by local people, and the vernacular name of each ethno-species was recorded. Then, these use reports were classified into six use categories. All these plants were native wild plants. In these plants, *Arisaema utile*, *Sorbus cuspidata* and *Elaeagnus umbellata* have been introduced into home gardens by local people. Following the description of the Sherpa people, we articulated a collection calendar for WEPs. The Sherpa collect WEPs throughout nearly the entire year, January and February being the exceptions.

**Conclusion:**

The collection calendar of wild edible plants reflects the wisdom of the Sherpa in terms of survival. The Sherpa cleverly survive the food shortage periods by harnessing the phenology of different species. In general, WEPs can provide the Sherpa with seasonal carbohydrates, nutrition, healthcare supplements and other products and services necessary for survival, which is likely why the Sherpa choose these plants.

**Supplementary Information:**

The online version contains supplementary material available at 10.1186/s13002-021-00464-x.

## Background

Food shortages are a serious global problem, especially in the less developed and remote regions of Asia and Africa. Climate change and extreme weather have increased the vulnerability of local farmers and herdsmen exacerbating the food shortage problem in these regions [1].The latest estimates show that nearly 690 million people (8.9% of the global population) are undernourished, with the largest number of hungry people located in Asia, prior to the 2019 COVID-19 pandemic [2]. The global pandemic of COVID-19 will negatively impact worldwide food systems [3].

Wild edible plants (WEPs), which are identified as non-cultivated and non-domesticated plants for food, could provide many products and services to the daily lives of local people in rural areas [4–11]. WEPs play a significant role as supplements to treat food shortages, when normal food supplies decline [10–13]. Additionally, WEPs can have healthcare [14] and nutrition benefits [15] for local communities. Local people can also earn extra income by selling WEPs [10]. As a part of a traditional expression, the traditional knowledge of WEPs can be lost along with the loss of traditional culture [16]. WEPs are considered an effective way to understand local knowledge and culture as they relate to biodiversity. In some remote and underdeveloped areas, WEPs are still an important source of plant products, such as vegetables, fruits, and starch to support daily needs [4–13]. Even in some urban areas, WEPs remain indispensable sources of vegetables and fruit [17–19]. Some starch-rich WEPs could help people in remote areas fight hunger in times of food shortages [20]. Furthermore, many rural people in developing countries face annual food shortages every year between the seasonal depletion of grain stocks and the next harvest [21]. WEPs are an important part of the daily diet in many remote and underdeveloped areas, which is especially obvious during seasonal food shortages [22]. Therefore, WEPs are vital in underdeveloped and remote areas.

The Mount Everest (Jo-mo-glang-ma-gang-ri or Qomolangma) area is the world’s highest mountain nature reserve. It is one of the world’s biodiversity hotspots, covering 36,000 km^2^ of the Himalayas [23]. The core region of the Mount Everest Reserve is one of the most remote and underdeveloped areas in both China and the world, sentailing that the livelihoods of native people heavily depend on natural resources [24]. Chenthang Township is located in the core area of the Mount Everest Reserve, and until recently, there was no driveable road linking the township to the outside [25]. As a result, people living in this area have to face rugged terrain, and the channels for the exchange of products and knowledge between the local community and the outside world are blocked. The Sherpa comprise a cross-border ethnic group living on both sides of the Himalayas, mainly in Nepal (Solu, Khumbu Canyon) and Tibet, China (Mount Everest National Nature Reserve) [26, 27]. Sherpa are known as “the Porters of the Himalayas”, because they work for mountain expeditions as guides and porters.

Some previous ethnobotanical studies of the Sherpa people focused on traditional edible plants and the utilization of traditional medicinal plants in central and eastern Nepal as well as the Indian state of Sikkim [28–31]. These studies showed that the Sherpa had accumulated a wealth of traditional knowledge about WEPs and medicines from the surrounding living environment. Furthermore, most of the studies in this area focused on its biodiversity, few discussed the correlation between local people and biodiversity, such as knowledge of local WEPs [24, 32]. There are also many studies regarding the WEPs of Tibetan-speaking ethnic groups in China [33–35]. To date, however, there is a lack of ethnobotanical studies concerning the Sherpa people in China. Therefore, it is necessary to investigate the WEPs and the related local knowledge of the Sherpa in China.

Local people living in rural and remote areas usually supplement their daily diet by using WEPs and have a wealth of accumulated plant knowledge. The ecological conditions and cultural significance of WEPs seem to affect local people’s knowledge and consumption of them [36]. The diversity and function of the plants in the local environment are closely related to the local people’s understanding of the surrounding plant world. Therefore, the core purpose of this research is to investigate, collect and record the WEPs and their local knowledge and functions in the Sherpa community. The ultimate goal is to understand why the Sherpa people chose certain plants over others.

Specifically, the purpose of this study was to investigate, collect and record the traditional use of WEPs and relative local knowledge accumulated by the Sherpa people in Tibet, China. The present study aims to answer the following questions: (1) What plants are consumed by the Sherpa people, and how can they be prepared? (2) What plants are important to the local community, and why? (3) What functions did these plants provide? (4) Why did the Sherpa people choose these plants?

## Material and methods

### Study sites

**Fig. 1:**
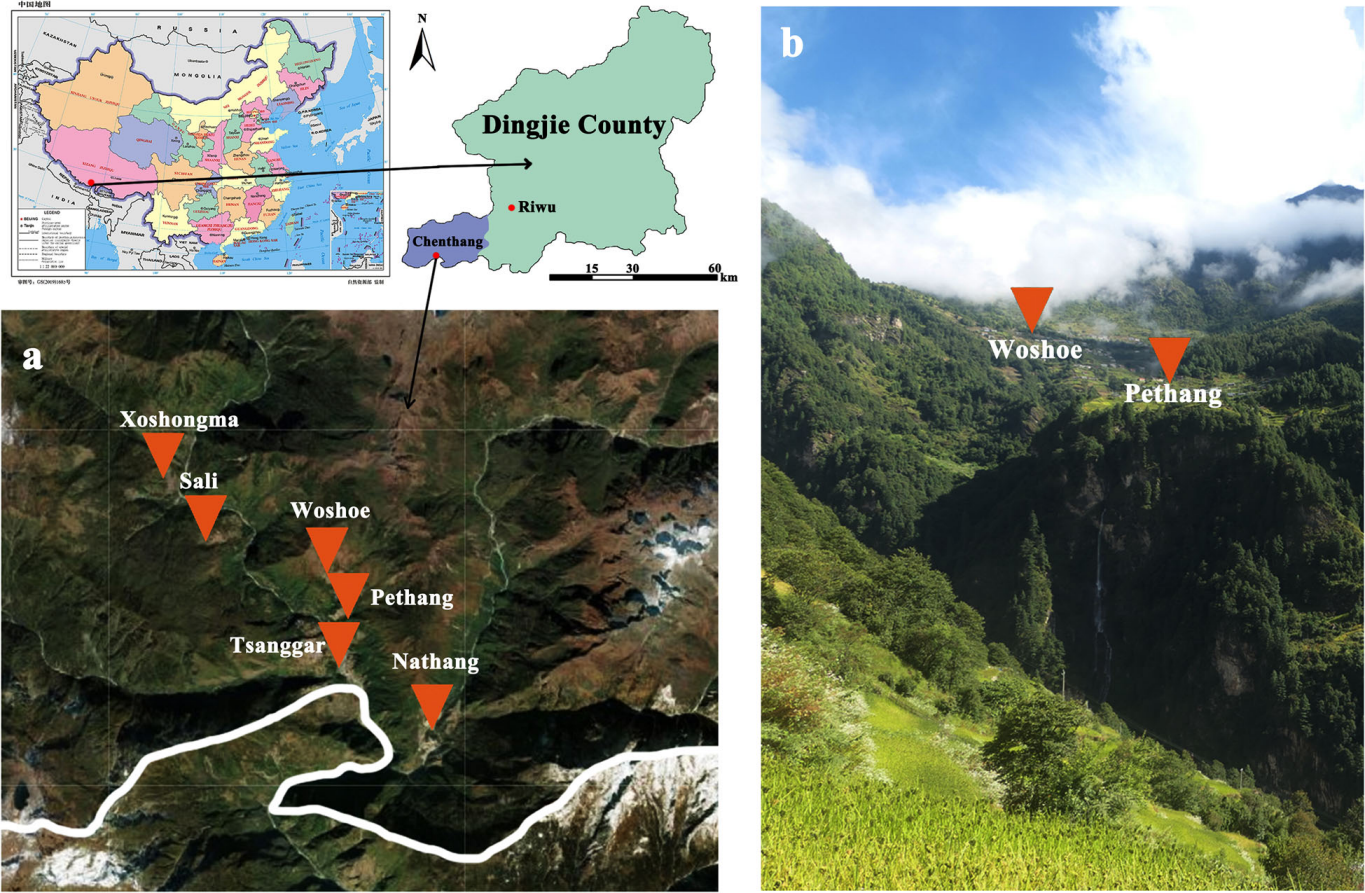
The location of study communities. Notes: **a** A satellite image of six villages in Chentang Town. **b** The natural landscape of Pethang Village and Woshoe Village, taken by Xiaoyong Ding

Chenthang Township, which is the only Sherpa ethnic township in Tibet, China, is located in the Mount Everest National Nature Reserve of the southeast Qinghai-Tibet Plateau on the border between China and Nepal (Fig. 1). The total area is 430.62 km^2^. There are 133.4 km^2^ of virgin forest, and the forest coverage rate is 98%, while the arable land area with a slope above 45° is only 59.87 ha. Based on 2016 demographics, there were 2387 herdsmen in 513 households in the town, including 2362 Sherpa, accounting for 99% of the total number [37]. The elevation of Chenthang Town ranges from 2040 to 5500 m. The annual frost-free period is approximately 200 days, and the annual average rainfall is over 1000 mm. The annual average temperature is 13.76 °C, and the annual extreme minimum temperature is 1 °C. Due to the effect of warm moist air flows in the Indian Ocean, Chenthang Town has a subtropical monsoon climate. The materials going in and out of Chenthang Town depend on manpower and livestock because of geographical limitations. Even now, Chenthang Township does not have a vegetable marketplace. Wind damage [38], hail damage [39], earthquakes [40] and other natural disasters have caused serious damage to farmland and pastures in the Xigaze area. Moreover, Xigaze City was one of the three main battlefields for poverty alleviation in the Xizang Autonomous Region [41].

The Sherpa language is a dialect of the Dbus-gtsang Tibetan language, which is written in the Tibetan alphabet [30]. Traditional Sherpa houses are made of timber or bamboo, and the Sherpa people live in sheds while grazing animals. The houses are built in a special form, usually with herringbone roofs, surrounded by thick walls made of stones and covered with thin planks. The main local crops are finger millet, winter wheat, corn, buckwheat, potatoes and some others. In addition, the stable food is Tsamba made of finger millet. The main economic activities are logging and harvesting medicinal plants, which are either sold for cash or traded for other goods and services. In the 1990s, people in Chenthang exchanged their daily necessities in Riwu town 100 km away. Carrying special products such as timber, medicine and bamboo baskets, they had to walk from Chenthang to Riwu Town for approximately 5 to 7 days.

### Field work and data collections

The field work was carried out within the six Sherpa communities of Chenthang Township (Table 1), from September 2019 to August 2020. First, we visited the township committee to obtain the field work permission. We explained our purpose to committee leaders and asked for help, which mainly included obtaining local guides, introducing us to local villagers and other necessary fieldwork assistance. The WEPs and related local knowledge were collected through semistructured interviews and direct observations. All of the fieldwork was carried out with informed consent. Because of the relative backwardness of the educational conditions, most local people, especially aged community members could not communicate fluently in the mainstream Chinese language. Therefore, the field work was performed with the assistance of local guides. The informants of semistructured interviews were selected randomly from the local community members, and we deliberately chose older people as the priority informants. Semistructured interviews were used to obtain information regarding the local knowledge of WEPs, which was provided by local people. The Vernacular names, life forms, usable parts, use, collection seasons, economic values and cultural significance of the plants were recorded. Direct observation was used to record the image and video data of the local uses of WEPs with a digital camera. Moreover, the voucher specimens were collected. The semistructured interviews were performed based on the following questions:

1. *Would you mind listing some wild plants you often consume, and which of them are edible?*
2. *Where are you collecting them, and when are their harvest seasons?*
3. *For these wild edible foods, could you tell us how to cook them for food?*
4. *In addition to foods, do you use them for other purposes? And what are these purposes? Could you tell us some traditional/old story or legend about these plants?*

**Table 1:** Characteristics of informants

Characteristics	Number	Percentage
Communities		
Tsanggar	12	15.4
Nathang	10	12.8
Xoshongma	14	17.9
Woshoe	16	15.4
Pethang	20	23.1
Sali	12	15.4
Gender		
Female	33	42.3
Male	45	57.7
Age		
Below 20	4	5.1
21–40	19	24.4
41–60	38	48.7
Above 60	17	21.8

### Information document and plant specimen identification and preservation

During the interview, we used a portable notebook to record information and then organized it into an Excel sheet (Microsoft Corporation, http://www.microsoft.com/) in a unified format, with “one plant to one local name” as a basic unit. We attempted to record all the information provided by the informants in an Excel sheet. All interviews were conducted in the Sherpa language, which was translated into Mandarin by local guides. The vernacular names of the plants were recorded in the Sherpa language which was written in the Tibetan script and its Latin form [30, 42].

During the field survey, with the help of local guides, we collected plant specimens based on the principle of one plant with one vernacular name. Digital photos were also taken to later identify the scientific taxa of plants. The collection of the voucher specimens was obtained, and the photographs of the photos were taken with the permission of the informants and the local community management department. Specimens were identified and stored in the herbarium of Kunming Institute of Botany, Chinese Academy of Sciences (KUN). Due to objective factors, we did not collect a voucher specimen of *Cardamine purpurascens*. However, this specimen was identified based on the video taken by the Sherpa (see Additional file 1). The identification of plant species was followed the Flora of Xizang and Flora of China [43, 44]. The proofreading of the Latin name of plants was based on The Plant List [45].

### Data analysis

We adopted the Use report (UR) and Cultural Important Index (CI) as ethnobotanical indices.

All local use and knowledge information are organized into a “use report” list for quantitative analysis according to three variables: information reporter, used plant and used method [46, 47]. A use report (UR) is the specific use of an ethno-species cited by an informant. In this study, all the scientific taxa of plants were consistent with those of the ethno-species.

The cultural important index (CI) was defined as the sum of the percentage of respondents who mentioned various uses for a certain useful plant. In this study, we used CI to evaluate the importance of WEPs in the daily diet of the Sherpa people. Additionally, CI considers the various uses of each plant, and the dissemination of knowledge (for each use category of each plant). In other words, the diversity of the plant’s uses and degree of recognition of the informants to each use category were included. It can evaluate the comprehensive utilization value of each useful plant. When u = 1, i.e. When the plant uses only one category, the CI and RFC index are equal in value, but they refer to different meanings [46]. CI is calculated by the following formula:

$$ CI=\sum \limits_{u={u}_1}^{{}^u NC}\sum \limits_{i={i}_1}^{{}^iN}\frac{UR_{ui}}{N} $$

To evaluate the comprehensive utilization value of each edible plant, we calculated the CI value. In addition, we calculated CI values for different use categories of each plant to understand the acceptance of the unique use category of a particular plant.

## Results

### Characteristics of informants

We interviewed 78 people individually, including 45 males and 33 females, from the six communities of Chenthang Township (Table 1). The age range of the informants was from 12 to 95 years old.

### Diversity of the wild edible plants

In total, we collected 84 edible species, including 79 angiosperms and five pteridophytes, belonging to 65 genera in 41 families (Table 2). The results showed that the most frequently mentioned families are Rosaceae (10 species), followed by Urticaceae (eight species) and Ericaceae (five species). Asparagaceae, Berberidaceae and Brassicaceae have four species. Magnoliaceae have three species. Eleven families have two species. The remaining 24 families have only species each. At the genus level, the most common genus was Rubus, followed by Vaccinium, Polygonatum, Urtica and Schisandra. Of the taxa, 46 were herbaceous plants, 17 were shrubs, 13 were trees and 8 were lianas (Table 2). All of these plants are native wild plants. In these plants, *Arisaema utile*, *Sorbus cuspidata* and *Elaeagnus umbellata* have been introduced into home gardens by local people.

**Table 2:**
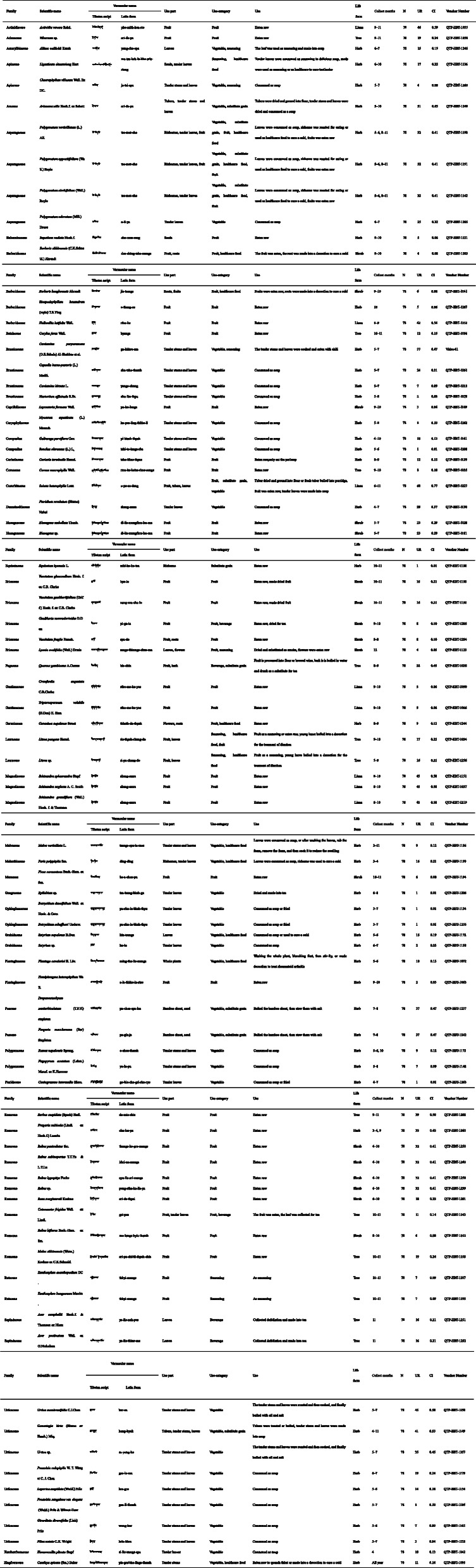
Wild edible plants in Chenthang Township

### WEP categories

These species were identified into 78 ethno-species by local people, and the vernacular name of each was recorded. In this study, we interviewed a total of 78 informants who provided us with 1199 use reports. These use reports were classified into six use categories (Table 3). The most frequent use category was “fruit” (485 use reports), followed by “vegetable” (451 use reports), “substitute grain” (102 use reports), “healthcare food” (78 use reports), “seasoning” (36 use reports) and “beverage” (47 use reports).

**Table 3:** Use report and use categories

Criteria	Use categories	Number of species	Use report (UR)
Plants material what were used to cook dishes (including making salads directly with raw plant material)	Vegetable	38	451
Fruits that were only eaten when they were ripe, similar to apple, pear and strawberry	Fruit	41	485
Not only edible plants, but could also be used by local people to treat diseases	Healthcare food	14	78
Plants that could be used as a direct starch supplement (e.g., tuberous or rhizome of some plants) or processed into starch (e.g., acorns)	Substitute grain	10	102
Plants that could be added to dishes or soups to increase the flavor of food	Seasoning	9	36
Plants that could be processed into home-made liqueurs or alcoholic beverages and processed into herbal teas	Beverage	6	47

### The most popular WEPs

In total, we collected 84 plant taxa, and 50 of them exceeded 10 use reports. However, there was only one use report for six plants and 10 plants with between two and five. The WEP CI values ranged from 0.01 to 0.77. According to the URs and CI values of ethno-species, the top five popular ethno-species were “o-pe-se-dang” (*Solena heterophylla*, CI = 0.77), “sri-da-pa” (*Arisaema utile*, CI = 0.65), “phe-mith-hen-rto” (*Actinidia venosa*, CI = 0.59), “skeng-smra” (*Schisandra grandiflora*, *S. neglecta*, *S. sphaerandra*; CI = 0.58), “bur-sa” (*Urtica membranifolia*, CI = 0.58) (Table 4). These plants have been used by the Sherpa for a long time, and most of them still are. For example, from May to July every year, the Sherpa women continue to travel together to collect the tender stems and leaves of *A. utile* and dry them naturally for winter consumption.

**Table 4:**
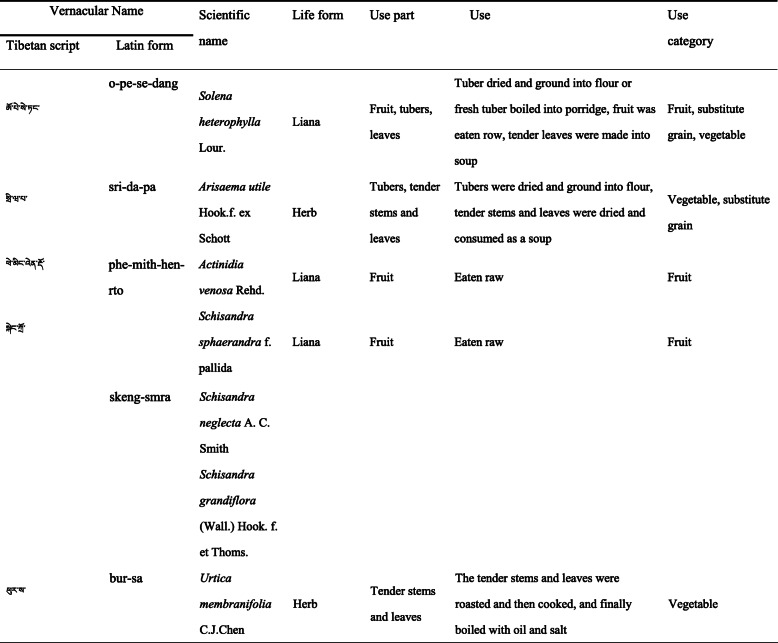
The top 5 popular ethno-species

### Collecting calendar of WEPs

**Fig. 2:**
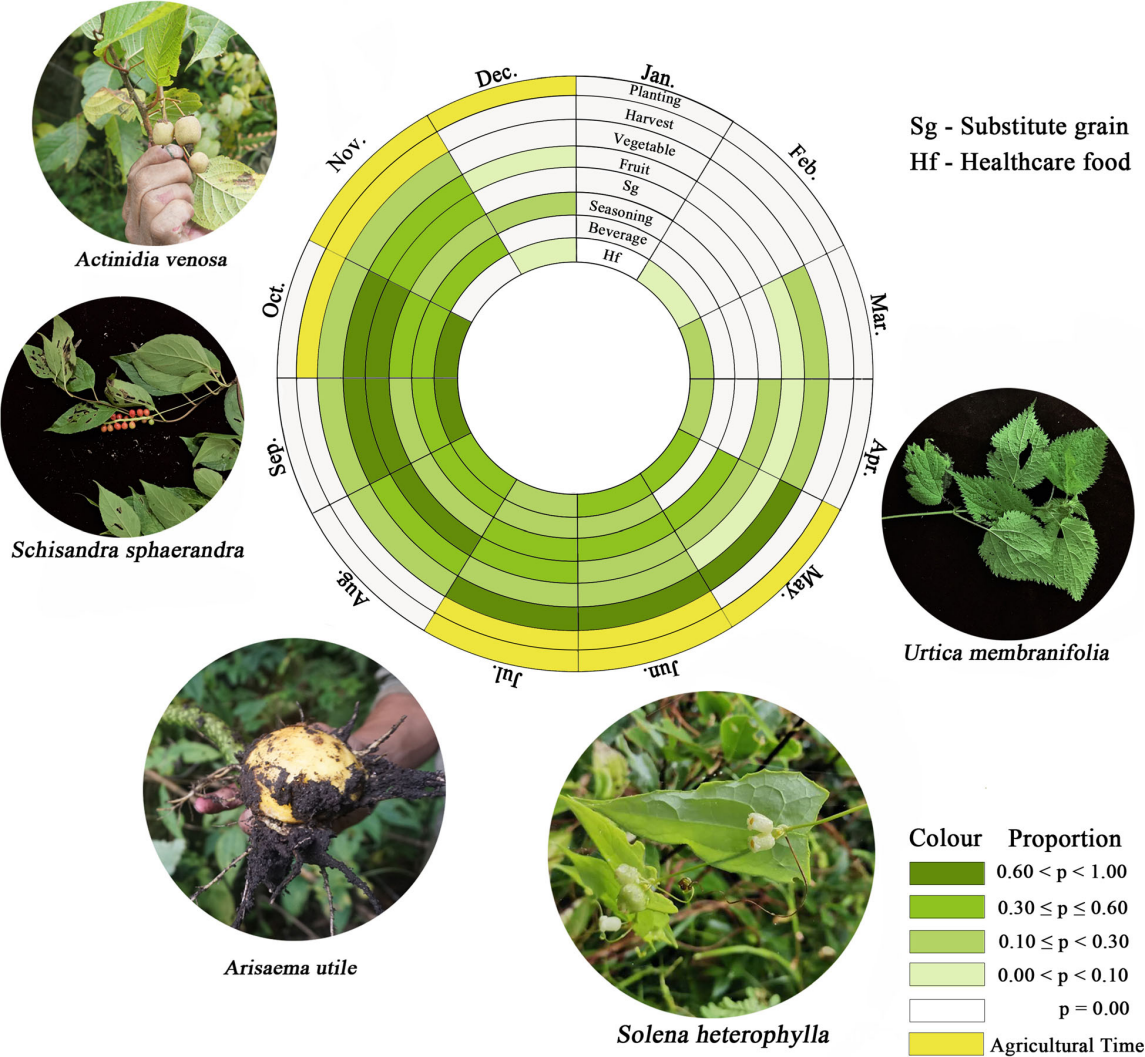
Collecting calendar of WEPs in Chenthang Town. Notes: (1) The shade of green indicates the percentage of the number of species collected for a single purpose, among the total number of species for that purpose, in each month. (2) Yellow indicates the time of the Sherpa’s agricultural activities

Implementing local people’s description, we recorded the farming season and collection time for each plant and drew a collecting calendar of WEPs (Fig. 2). There are 8 circles in Fig. 2, corresponding to six use categories and two agricultural activities. The circle is divided into 12 equal parts, and one part represents 1 month. The shade of green indicates the percentage of the number of species collected for a single purpose, among the total number of species for that purpose, in each month. The deeper the green, the more plant species are collected. The Sherpa collect WEPs throughout almost the year, except for January and February. The most concentrated period of wild vegetable collection was from May to July, and wild fruit was mainly from September to October. Subsistence grain was mainly collected from July to November. Most of the used sections of spice and healthcare plants are roots or seeds, which are collected from July to November (Fig. 2).

The top 5 ethno-species are displayed outside the circle. The larger the picture is, the higher the frequency of the ethno-species being cited. The position of the picture represents the time when the plants were collected. *Solena heterophylla* was collected from June to November. *Arisaema utile* was collected from May to October. *Actinidia venosa* was collected from September to November. *Schisandra sphaerandra* was collected from August to November. *Urtica membranifolia* was collected from May to July.

## Discussion

### The important WEPs

Based on the information provided by the informants, we carried out a quantitative analysis, and the results showed that there were some WEPs that are very important to the Sherpa people. These plants are collected seasonally and used for multiple purposes or mentioned frequently (Table 3).

“O-pe-se-dang” (*Solena heterophylla*) was the most frequently cited of the recorded plants. Local people collect *S. heterophylla* for multiple purposes. Young leaves could be collected and cooked as vegetables, and mature fruits could be collected as seasonal fresh fruits. It should be highlighted that the starch-rich tuber of *S. heterophylla* is one of the important alternative grain sources for the Sherpa people during the periodic and aperiodic famine. *S. heterophylla* is also used by other indigenous tribes settling in the Himalayan region. Our previous study showed that the species was consumed as a fruit by the Monpa in eastern Himalaya [48, 49]. In addition, *S. heterophylla* is also used in local medicine. The Sherpa people in Nepal consume small amounts of its fruit or tuber cream to treat throat infections associated with fever and often ate ripe fruit to ensure that abdominal ulcers are cured [30]. Traditionally, the Hani ethnicity took the tuber of the species in decoction to treat stomachache in southern Yunnan, China [50].

“Sri-da-pa” (*Arisaema utile*) was the most frequently cited substitute grain (CI_Grain_ = 0.53). From August to November, tubers were collected and prepared as substitute grain to treat seasonal food shortages. The tender stems and leaves of *A. utile* are local wild vegetables, and they were dried naturally and stored for cooking in winter or during major festivals. Sometimes, the Sherpa dig *A. utile* up and plant it in the home garden so that it can be eaten at any time. The fresh fruits of Araceae plants are local herbal medicines to treat flatulence and gastrointestinal discomfort in southern western Ghats, Tamil Nadu, India [51]. The Khampa Tibetan people stir fried young leaves of *Arisaema erubescens* as a supplementary vegetable and used tubers to relieve cough and treat haemoptysis and pneumonia in Northwest Yunnan, China [52].

“Phe-mith-hen-rto” (*Actinidia venosa*) was the most mentioned wild edible fruit (UR = 46, CI_Fruit_ = 0.59). This plant is an important and easily available wild edible fruit for the Sherpa to supplement vitamin C from September to November. Previous studies also showed that many species of Actinidia are rich in vitamins [7, 48, 53].

“Skeng-smra” (*Schisandra grandiflora*, *S. neglecta*, *S. sphaerandra*) was another very important wild edible fruit that supplements nutrients seasonally, with 45 use reports. The local name of “skeng-smra” has three plant taxa. Sometimes, locals collected a large bag of “skeng-smra” and ate it when the family rested while working on the farm. In China, *S. chinensis*, a traditional Chinese medicine, has been widely used in medicine and health food in recent years. This plant contains a variety of chemical components for the treatment of the central nervous system, cardiovascular and cerebrovascular system, hypoglycaemia, liver protection and other aspects of potential pharmacological activities [54–56].

“Bur-sa” (*Urtica membranifolia*) was the most mentioned wild edible vegetable (UR = 45, CI_Vegetable_ = 0.58). It is an important source of vegetables for the Sherpa people from May to July every year and is often processed into a paste and eaten with potatoes.

### The functions of WEPs collected seasonally by the Sherpa

WEPs are still an important part of the daily diet in many remote and underdeveloped areas, which is especially obvious during seasonal food shortages [22]. Due to the different growth cycles of plants in nature, the collection of wild plants in many places has clear seasonality [10, 53]. The acquisition of WEPs was closely related to the shortage of cultivated food resources [11]. When normal food supply mechanisms were destroyed by events, such as famine, wild food was very important for the poor and the landless. The growth of crops takes time, and wild vegetables can grow quickly, which enhances the resistance of the local food system [4].

**Fig. 3:**
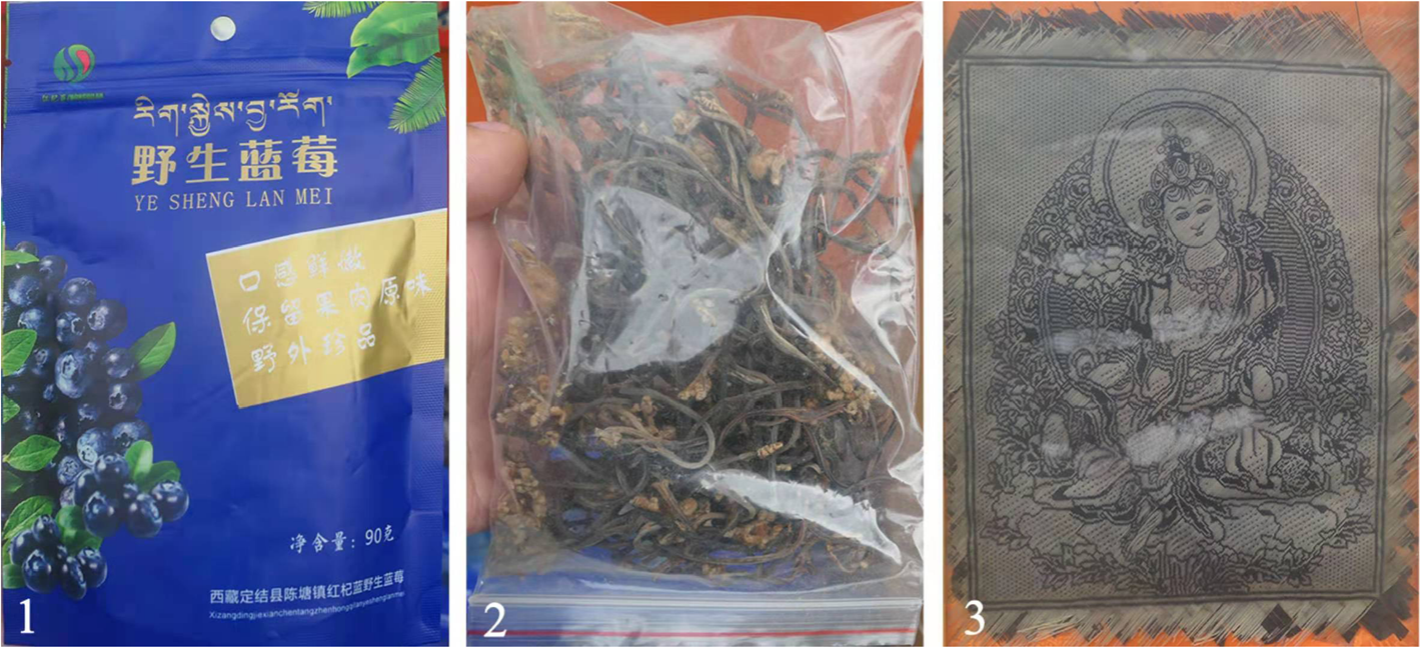
Three wild economic plant products. **1** Wild dried *Vaccinium glaucoalbum* and *V. gaultheriifolium* fruits. **2** Dried young *Pteridium revolutum* leaves. **3** Bamboo woven Thangka

In the past, the Sherpa in Chenthang hunted for food; thus, they formed a “hunting culture” suitable for their economic life. Previously, there was no land, and the agricultural management method was slash and burn cultivation. The mayor of Chenthang Town told us that in 2015, there were 2321 people, and the annual output of grain was 165.5 tons. The average annual grain output per capita is approximately 71.3 kg, which is far below the internationally recognized food safety standard of 400 kg per capita. Moreover, the native fruit trees only have *Amygdalus mira* and *Juglans regia*, which have low productivity and seasonal restrictions. Due to the large altitude gradient, a diverse climate gradient is formed in Chenthang, which creates a diversity of wild plants. Therefore, WEPs are chosen by the Sherpa people (and there is no other choice) as an important source of nutrients such as vitamins, minerals and trace elements. The survey results showed that the diverse WEPs collected at different times provided different functions and services for the Chentang Sherpa people at specific periods (Fig. 3).

#### The self-service “natural carbohydrate factory” for the Sherpa

WEPs play an important role in supplementing staple food under normal circumstances [57]. From January to March and from August to November, there was a period of food shortage. “When there was a shortage of food, from January to March, we collected ‘bis-chin’ (*Quercus gambleana*) to be processed into flour for consumption. From August to November, we collected ‘sri-da-pa’ (*Arisaema utile*) to be processed into flour for consumption”, the oldest woman described.

Chentang Town is under heavy snow from January to March, and the Sherpa people mainly consume stored grain to survive. Therefore, every August and September, Sherpa women collect the nuts of *Q. gambleana*, process them into starch and store them and consume this in winter. Locals told us that the meal made from the starch of *Q. gambleana* fills them up all day. In 2009, Liu tested the starch content of *Quercus ciliaris* and *Q. glauca*, the former content was 20.9%, and the latter was 26.2% [58]. In Asia and the Mediterranean basin, *Fagaceae* plants are widely used for cooking, pasture, and building materials, and the most commonly used parts are nuts [20, 48, 59, 60]. We can therefore speculate that the genus *Quercus* has a certain content of starch.

After July, the staple food of the locals was potatoes almost every day. From August to November, *A. utile* was collected, processed and consumed. In Nepal, the tuber of *Arisaema intermedium* is an important wild supplementary food that can be used as a condiment under normal circumstances and as an emergency food ration during poor harvests and food shortages [61]. In dried tubers of *A. elephas*, *A. yunnanense* and *A. erubescens*, the starch content was 15.37%, 61.60% and 52.91%, respectively, among the three kinds of starch, and the amylopectin content ranged from 29.1 to 32.0% [62]. The starch content of *A. utile* needs further research.

In addition, other WEPs are also used as seasonal alternatives to grain consumption. The tubers of *Solena heterophylla* were dug out, mashed, boiled into porridges or dried and then ground into flour to make Tsampa. In winter, the Sherpa people also baked the rhizomes of *Polygonatum verticillatum*, *P. oppositifolium*, *P. cirrhifolium* and *Paris polyphylla* as food. Here are a few lesser-known but not missed alternative grain plants, namely, the rhizomes of *Equisetum hyemale* and bamboo seeds that are also consumed as a source of starch. The rhizome of *E. hyemale* was first recorded as an edible plant. These starch-rich plants can be considered to be a self-service “natural carbohydrate factory” to help them survive food shortages.

#### The natural nutrition supply station for the Sherpa

WEPs are an important alternative source for people in remote and poor areas to obtain nutrients and biologically active compounds, such as vitamins and minerals, in addition to cultivating vegetables and fruits [63]. In remote areas with inconvenient transportation, wild vegetables are the main source of vitamins for local people, especially women and children [64].

Wild vegetables, regarded as healthy and beneficial foods, are rich in trace elements, cellulose, flavonoids, saponins and vitamins [65]. From May to July each year, a large number of wild vegetables are collected and consumed, some of which are consumed as soon as they are collected, while others are processed and stored. For example, the tender stems and leaves of *A. utile* and the tender leaves of *Polygonatum odoratum* and *Pteridium revolutum* were naturally dried and stored for consumption when there were no vegetables in the winter or there were large festivals such as weddings.

Some studies have shown that WEPs contribute nutrients to humans and revealed the nutrients contained in these plants. Yang analysed dry samples of *P. revolutum* and found that it contained 29.42% protein, 7.4% soluble sugars, 16.27% crude fibre, 1.05% crude fat, a potassium content of 3772 mg/100 g, a magnesium content of 377 mg/100 g, a calcium content of 215 mg/100 g, an iron content of 5.589 mg/100 g and a manganese content of 10.420 mg/100 g, which were much higher than cabbage and spinach [66]. According to the determination, the leaves of *Urtica laetevirens* from May to July contained 23.73 to 34.75% crude protein and 17 kinds of amino acids. Aspartic acid and serine reached the maximum value (3.33%) in June, and histidine and threonine reached the maximum value (2.52%) in May. Minerals and vitamins had higher levels from May to July, and then showed a downward trend [67]. Analysis of the nutritional components of *Schisandra chinensis* showed that the crude protein content of *S. chinensis* ranged from 10.67 to 11.69% after entering the mature period, while that of Schisandrin ranged from 2.63 to 5.47 g/kg. This plant contains 7 amino acids and essential elements (P, K, Mg, Fe, Zn and Cu) that are necessary for the human body [68]. Kiwifruit is rich in minerals and vitamins, which can enhance human immunity, digestion and metabolism, and improve nutritional status [69].

People in different regions of the world are still consuming WEPs, but the important contribution of these plants to the human diet is still not recognized in developed regions [63]. Research on wild edible vegetables could clarify how important WEPs are to the rural dietary structure while also providing potential trace element resources for the urban population [70]. The further scientific utilization and development of WEPs would help provide protection for human nutrition, especially among people in remote areas.

#### The free “herb shops” for the Sherpa

WEPs usually fulfil the function of medicine and food. Wild vegetables not only provide minerals and vitamins but can also be used as medicine for food security and health [10, 71]. For example, it was found that WEPs have anticancer (especially breast and stomach cancer), anti-inflammatory, antioxidant and anti-diabetic effects through the study of 56 kinds of wild vegetables [72]. In our study, 11 of the WEPs we collected had medicinal functions. The methods of preparation, local names, parts used and ailments treated with the treated plants were recorded. The results are consistent with the statistics of common and endemic diseases—dysentery, intestinal parasitic diseases, arthritis and so on—in Chenthang town by Xigaze City and Dingjie County health station in Xizang Autonomous Region [73, 74]. There is another interesting phenomenon. *Polygonatum verticillatum*, *P. oppositifolium*, *P. cirrhifolium* and *Paris polyphylla* are traditionally used as edible plants instead of herbs in Chenthang. The leaves of these plants were consumed as soup, and rhizomes were roasted in winter to supplement starch. With the opening of Chenthang, the Sherpa people obtained some medical knowledge from tourists and drug dealers. Now, they know how to use the roots of these plants to cook a decoction that treats the common cold. The exchange of information affects the dissemination of knowledge, to some extent.

#### Improve livelihoods and help local communities reduce poverty

Local people could earn extra income by selling some of the economic WEPs [10]. Local people often sell WEPs to urban residents and tourists in the market to increase their income [75]. Because of economic and social development, Chenthang Town welcomes tourists from all over the world with an open attitude. With the help of the Chinese government, some wild vegetables and fruits, such as *Pteridium revolutum* and *Vaccinium glaucoalbum*, were processed into products by the Sherpa people and sold to tourists (Fig. 3). Moreover, the long-standing bamboo thangka of the Sherpa was also made into products. Although bamboo thangka could be produced only 3 months in a year, there was a total profit of 150,000 yuan on it in 2016, which helped 15 poor households move out of poverty [76]. However, while wild plants increase local income, their excessive collection has caused a certain degree of damage to the local plant community. In recent years, drug dealers have come to Chenthang Town to purchase these medicinal plants in large quantities, resulting in their being overharvested. Studies have shown that the huge market potential and the uncontrolled collection of medicinal plants have led to the disappearance of certain herbs from their natural habitat [77]. The unsustainable collection has also led to a decrease in the population of some edible plants with high market prices [52]. Not only WEPs are threatened, but the local knowledge associated with them is also at risk. While developing the economy, we must pay attention to sustainable development based on the local ecological environment and biological resources.

#### Cultural implications

Many WEPs also have cultural value. Some are used in religious and cultural activities and considered sacred. Some vegetables also have a certain social and cultural carrier function in addition to their collection and donation activities, functioning as a link to promote internal communication in the community [78]. The Sherpa people have a long history of bamboo weaving. With the rise of stainless steel and plastic appliances, bamboo weaving appliances have gradually lost their advantages, so bamboo weaving skills are facing a crisis of loss. These skills combine traditional bamboo weaving techniques and Thangka painting techniques to create bamboo weaving thangkas. The bamboo slices processed by them are as thin as a cicada’s wing and as tough as pampas grass. They are covered in clearly visible text. Weaving these thin bamboo slices into thangkas not only retains the true qualities of thangkas, but also adds new agility to them. The act is extremely artistic and is the inheritance of national culture. The utensils used in Sherpa homes, such as fruit baskets and fruit plates, are all made of bamboo [76]. Therefore, bamboo weaving has become a cultural symbol of Sherpa. In our research, there were 26 kinds of plants with two or more use categories, further demonstrating the importance of these plants both for local survival and as a cultural heritage.

The diversity of the facets and methods for using WEPs also highlight the unique food culture of the Sherpa, to a certain extent. The purpose of local people using wild plants varies according to their hierarchy of needs [11, 51, 79]. Plants are usually used by people for various purposes, such as food, medicine, fuel, and economic income. In Chenthang, there is a very interesting phenomenon. For example, with *Solena heterophylla* in this study, the Sherpa only developed the function of food. However, thousands of miles away in Yunnan, the Hani people use *S. heterophylla* tuber decoction to treat stomachache [52]. The rhizomes of *Equisetum hyemale* were found to be eaten be the Sherpa for the first time. *Polygonatum verticillatum*, *P. oppositifolium*, *P. cirrhifolium* and *Paris polyphylla*, which are famous traditional Chinese medicines, are traditionally used as wild vegetables and substitute grain plants instead of herbs by the Sherpa people. Although studies have centred on the effects of different geographical conditions, vegetation types and cultures may also influence the use of wild plants in different areas [79]. In other words, people in different regions have strong regional and cultural characteristics that affect their selection and utilization of WEPs. Further research should focus on this issue.

### Detoxification and potential safety hazards

Although WEPs have many advantages, there are also some hazards. Wild plants often contain compounds toxic to humans, such as nitrates and oxalic acid [63], and excessive consumption of these may cause problems for human health. Staple foods that many people rely on require complex detoxification procedures, including prolonged cooking, roasting or leaching [80]. Therefore, it is necessary to be aware of the toxic compounds contained in these wild edible vegetables and to know the proper treatment process to remove poisons [81, 82].

Over a long period of time, the Sherpas have developed special methods to make their food safer. For example, the Sherpa call the process of removing tannins from nuts of *Quercus gambleana*, “Remove black water”. After the collected nuts are shelled and processed into large particles, they are wrapped in the leaves of the azalea and washed continuously with water. When the “black water” is removed, the large particles of nuts will turn yellow. “*If the harvested nuts are not processed, they will taste bitter. Furthermore, if you eat too much, you will get constipation*”, the locals told us. The reason may be that the tannins were not completely removed [83].

“Sri-da-pa” (*Arisaema utile*) is a multifunctional plant, and its tuber detoxification process is known to the Sherpa as “Squeeze the juice”. Fresh tubers are processed into large pellets and then wrapped in azalea leaves, after which a heavy stone is pressed on them. After approximately 15 days, no additional juice flowed out, and the sour smell emerged. The detoxification process was complete. During processing, if you do not take protective measures, your hands will be swollen, itchy or even peel. At this time, you need to apply some butter. The cause of the adverse reaction may be that the calcium oxalate needle crystals contained in the Araceae had not been removed cleanly [84].

However, local knowledge alone is not enough to manage food safety. Some WEPs still have certain side effects after being processed. For example, fresh tubers of *Solena heterophylla* are often processed into porridges by the Sherpa people. Yet, even when heated and stewed for a long time, locals still feel a chin itch and stomach upsets after eating the porridge. Therefore, applying modern scientific methods to detect and monitor the toxicity of these edible plants and raising public awareness of food safety based on scientific research could fundamentally improve this problem. Future research could certainly pay more attention to local knowledge of food safety.

## Conclusion

WEPs are an important source of plant products such as substitute grains, vegetables and fruits to support daily lives in Chenthang Township, one of the most remote and underdeveloped areas in both China and the world. These diverse WEPs provide many functions and services for the Sherpa to survive. In total, the study examined 84 species of WEPs used by the Sherpa, belonging to 65 genera in 41 families. The local name of each Botanical taxa was recorded. Sometimes, a local name has multiple Botanical taxa. The results showed that the most frequently mentioned family by the informants was Rosaceae, followed by Urticaceae. According to the URs and CI value of ethno-species, the top five popular plants were “o-pe-se-dang” (*Solena heterophylla*), “sri-da-pa” (*Arisaema utile*), “phe-mith-hen-rto” (*Actinidia venosa),* “skeng-smra” (*Schisandra grandiflora*, *S. neglecta*, *S. sphaerandra*), “bur-sa” (*Urtica membranifolia*). We interviewed 78 informants who provided us with 1199 use reports. These use reports were classified into six use categories. The most frequent use category was “fruit”, followed by “vegetable” and “substitute grain”. According to the description of the Sherpa people, we drew a collecting calendar of WEPs. The Sherpa collect WEPs throughout almost the entire year, except for January and February.

The collection calendar of WEPs reflects the wisdom of the Sherpa regarding survival. They cleverly survive the food shortage period by means of harnessing the phenology of different species in Chenthang Township that is virtually isolated from the outside world and have few land resources. In general, WEPs provide the Sherpa with seasonal carbohydrates, nutrition, healthcare supplements and other products and services necessary for survival, which is likely why the Sherpa choose these plants..

## Supplementary Information

**Additional file 1.**

## Data Availability

Please contact the corresponding author for data requests.
